# Electron Paramagnetic Resonance for the Detection of Electrochemically Generated Hydroxyl Radicals: Issues Associated with Electrochemical Oxidation of the Spin Trap

**DOI:** 10.1021/acsmeasuresciau.2c00049

**Published:** 2022-09-26

**Authors:** Emily Braxton, David J. Fox, Ben G. Breeze, Joshua J. Tully, Katherine J. Levey, Mark E. Newton, Julie V. Macpherson

**Affiliations:** † Department of Chemistry, University of Warwick, CoventryCV4 7AL, U.K.; ‡ Molecular Analytical Science Centre for Doctoral Training, University of Warwick, CoventryCV4 7AL, U.K.; § Department of Physics, University of Warwick, CoventryCV4 7AL, U.K.; ∥ Centre for Doctoral Training in Diamond Science and Technology, University of Warwick, CoventryCV4 7AL, U.K.

**Keywords:** electron paramagnetic resonance (EPR), electron spin resonance (ESR), electrochemical-EPR, spin trapping, dimethyl-1-pyrroline *N*-oxide (DMPO), hydroxyl radical, boron-doped diamond (BDD)

## Abstract

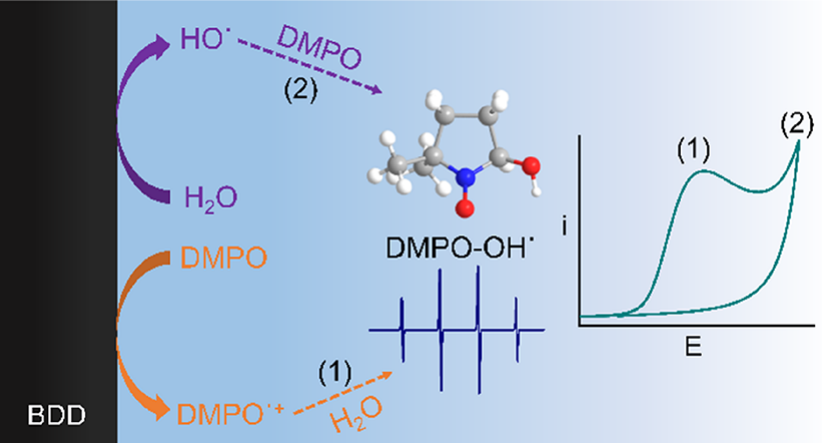

For the detection of electrochemically produced hydroxyl radicals (HO^·^) from the oxidation of water on a boron-doped diamond (BDD) electrode, electron paramagnetic resonance spectroscopy (EPR) in combination with spin trap labels is a popular technique. Here, we show that quantification of the concentration of HO^·^ from water oxidation via spin trap electrochemical (EC)-EPR is problematic. This is primarily due to the spin trap oxidizing at potentials less positive than water, resulting in the same spin trap-OH^·^ adduct as formed from the solution reaction of OH^·^ with the spin trap. We illustrate this through consideration of 5,5-dimethyl-1-pyrroline *N*-oxide (DMPO) as a spin trap for OH^·^. DMPO oxidation on a BDD electrode in an acidic aqueous solution occurs at a peak current potential of +1.90 V vs SCE; the current for water oxidation starts to rise rapidly at ca. +2.3 V vs SCE. EC-EPR spectra show signatures due to the spin trap adduct (DMPO-OH^·^) at potentials lower than that predicted thermodynamically (for water/HO^·^) and in the region for DMPO oxidation. Increasing the potential into the water oxidation region, surprisingly, shows a lower DMPO-OH^·^ concentration than when the potential is in the DMPO oxidation region. This behavior is attributed to further oxidation of DMPO-OH^·^, production of fouling products on the electrode surface, and bubble formation. Radical scavengers (ethanol) and other spin traps, here *N*-*tert*-butyl-*α*-phenylnitrone, *α*-(4-pyridyl *N*-oxide)-*N*-*tert*-butylnitrone, and 2-methyl-2-nitrosopropane dimer, also show electrochemical oxidation signals less positive than that of water on a BDD electrode. Such behavior also complicates their use for the intended application.

## Introduction

Free radicals are highly reactive species often with very short lifetimes. Electrochemistry is a powerful method for free radical creation, and as such, electrochemically generated free radicals have been used in a wide range of applications including electrochemical advanced oxidative processes (EAOPs)^1−3^ and electrosynthesis.^4−9^ One of the most widely studied electrochemically produced free radical is the hydroxyl radical (HO^·^) due to its very high oxidizing potential and the fact it can be electrochemically generated from water at high anodic potentials, eq 1.^10^
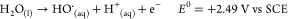
1

HO^·^ is considered the predominant species responsible for the degradation of environmental pollutants in EAOPs.^1,3^ In order to electrochemically produce freely available HO^·^ in solution, “non-active” electrodes are required, such as boron-doped diamond (BDD),^11,12^ which disfavor adsorption of HO^·^ on the electrode surface.

The most popular techniques for free radical detection are electron paramagnetic resonance (EPR) spectroscopy^13−15^ (also known as electron spin resonance (ESR) spectroscopy) and fluorescence spectroscopy.^16−18^ EPR offers low limits of detection as well as accurate identification of different free radical species through distinctive splitting patterns and is often used in combination with electrochemical experiments.^19,20^ Due to the extremely short lifetimes of many free radicals, particularly in aqueous solutions (e.g., HO^·^ in water has a lifetime of ∼ μs),^21^ EPR spectroscopy often requires the use of spin trap reagents to convert the free radicals into more persistent species, which are stable over the timescale of the measurement.

In EPR spin trap chemistry, the free radical (R^·^) reacts with the spin trap (ST) to form a longer-lived paramagnetic spin adduct (ST-R^·^), eq 2.
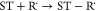
2

ST-R^·^ thus provides indirect detection of the free radical of interest. However, there are other possible pathways that can also lead to the formation of ST-R^·^ but do not require R^·^ as the starting species. These include inverted spin trapping,^22−25^ as shown in eq 3, where the spin trap loses an electron (by electrochemical/photochemical/chemical oxidation or ionization routes), making it very susceptible to attack by a nucleophile (NuH) and formation of ST-Nu^·^.
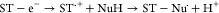
3

Also important is the Forrester–Hepburn mechanism^26−28^ (eq 4), which describes attack of a nucleophile (NuH) on the spin trap, followed by electron abstraction by an appropriate oxidizing agent. Equation 4 is facilitated by the presence of chelating metal impurities such as Fe^3+^,^29,30^ Cu^2+^,^30^ Ti^5+^,^31^ Au^3+^,^32^ and Tl^3+^,^33^ which can bind to the spin trap and promote easier nucleophilic attack.
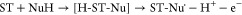
4

Equations 3 and 4 are especially problematic when considering spin trap detection of HO^·^ in an aqueous system. This is because water can act as a nucleophile (albeit a weak one), meaning there is possibility for forming the hydroxyl spin adduct (ST-OH^·^) via either electrochemical oxidation of the ST (eq 3) or direct attack of the ST by water (eq 4) followed by electron abstraction. While eqs 3 and 4 are well reported for the spin trapping of HO^·^ in biological media^27,34,35^ and for fluorescence detection of HO^·^,^36^ detailed studies are lacking for electrochemically generated and EPR spin-trapped HO^·^ free radicals in aqueous systems. This is somewhat surprising, especially as EPR is the “go-to” method for confirmation of the existence of electrochemically generated HO^·^.^13−15,37−39^ As the potential required to electrochemically generate HO^·^ in aqueous solution is so high (eq 1) and water is present in excess, concerns must be raised over the roles of eqs 3 and 4 in providing false positives for the EPR detection of spin trapped HO^·^ generated electrochemically.

In other non-electrochemical disciplines, the role of inverted spin trapping (eq 3) has been tackled by the addition of free radical scavengers such as ethanol, dimethyl sulfoxide, or ethyl acetate.^29,30,40^ If the spin adduct is formed as a result of spin trapping (eq 2), the addition of a free radical scavenger would compete with the spin trap for free radicals, leading to a significant reduction in spin adduct concentration. Furthermore, if the radical scavenger used contains an α-hydrogen (*i.e.*, a hydrogen on a carbon adjacent to a functional group), it is possible for the free radical to abstract the α-hydrogen and produce a radical with a different distinctive spin adduct compared to the spin adduct formed by eq 2. However, these methods fail to consider the implications of electrochemical oxidation of the scavenger itself.

EPR spin traps usually have either a nitroso- or nitrone- functionality, which generates a nitroxide free radical spin adduct when the free radical is trapped.^41^ Inclusion of such functional groups, however, make the spin trap prone to electrochemical oxidation.^22,42^ A very popular spin trap for electrochemically generated HO^·^ detection is the nitrone-based 5,5-dimethyl-1-pyrroline *N*-oxide (DMPO).^13−15,37,39,43^ The DMPO adducts possess a β-hydrogen enabling the discrimination of small radical species through the different observed splitting parameters.^44^

Even though the first report of the electrochemical oxidation of DMPO was published in the 1980s^42^ using a platinum (Pt) electrode in a non-aqueous solvent, the potential issues of using DMPO for HO^·^ electrochemical-EPR (EC-EPR) detection has still not been widely acknowledged in the EC-EPR community. Since then, to our knowledge, only one other study has investigated the electrochemical oxidation of DMPO in aqueous media, using Pt and titanium suboxide electrodes. A peak oxidative potential of +1.48 V vs SCE was reported using Pt.^45^ However, no EC-EPR data were shown at potentials where only DMPO oxidation occurs in order to verify the role of eq 3 in producing false positives in the EPR identification of electrochemically generated HO^·^.

In this paper, a comprehensive investigation of the electrochemical oxidation of DMPO in aqueous solution is given as well as a discussion of the products formed. The implications of DMPO oxidation on successful HO^·^ detection (formed via electrochemical means) in EPR spectroscopy is also explored. We also investigate the effect of adding a radical scavenger (ethanol) in an EC-EPR experiment. Finally, electrochemical characterization of other common EPR spin traps is performed, including *N*-*tert*-butyl-*α*-phenylnitrone (PBN), whose electrochemical characteristics could not be previously resolved using a Pt electrode, within the aqueous solvent window.^42^

## Experimental Section

### Reagents and Solution Preparation

Solutions were prepared using deionized water of ≥18.2 MΩ cm resistivity at 25 °C (Milli-Q, Millipore Corp.). All chemicals were used as received from the supplier. Stock DMPO (<98%, Enzo Life Sciences Ltd) was stored in a freezer at −18 °C prior to use and prepared at a range of concentrations (1–10 mM) in 0.10 M perchloric acid (HClO_4_; 70%, 99.999% trace metals basis, Sigma Aldrich) unless otherwise stated. To minimize migration and ohmic drop,^46,47^ in the linear sweep voltammograms (LSVs) a DMPO to electrolyte ratio of ≥1:10 was employed. The 15, 20, and 30 mM DMPO solutions were prepared in 0.15, 0.20, and 0.30 M HClO_4_, respectively. A solution of hexammineruthenium(III) chloride (Ru(NH_3_)_6_^3+^; 99%, Strem chemicals) was prepared at 1 mM in 0.10 M potassium nitrate (KNO_3_; ≥99.0%, Sigma Aldrich). To calculate spin adduct concentrations, the stable radical 4-hydroxy-2,2,6,6-tetramethyl-1-piperidine 1-oxyl (4-hydroxy TEMPO; 98+%, Alfa Aesar) was prepared at concentrations over a range of 500 nM to 100 μM in deionized water, to produce a calibration curve. Solutions of 10 mM *N*-*tert*-butyl-*α*-phenylnitrone (PBN; >99.5%, Sigma Aldrich), *α*-(4-pyridyl *N*-oxide)-*N*-*tert*-butylnitrone (POBN; 99%, Sigma Aldrich), and 2-methyl-2-nitrosopropane dimer (MNP; Sigma Aldrich) were also prepared in 0.10 M HClO_4_. EC-EPR experiments using ethanol as a radical scavenger were performed in an aqueous solution containing 5 M ethanol (99.9% absolute, VWR Chemicals) in 0.10 M HClO_4_. Finally, a solution of 0.10 M tetrabutylammonium tetrafluoroborate (TBAB; 99%, Sigma Aldrich) in ethanol was prepared.

### Electrode Preparation

For the large BDD electrode studies, a rectangle (1 × 7 cm) of BDD was cut from a 700 μm-thick freestanding BDD wafer with a boron dopant density of >10^20^ B atoms cm^–3^, *i.e.*, above the metallic threshold^48^ (Element Six, Electrochemical Processing grade).^49^ For EC-EPR measurements, both the front (as-grown) and back (nucleation) faces of the electrode were immersed in solution. The surface roughness for both faces of the electrode was measured by white light interferometry (WLI). The growth face had a RMS roughness of ∼10 μm, while the nucleation face had a RMS of 150 nm. The electrode was cut to size using a 355 nm Nd:YAG 34 ns pulse laser micromachining system (E-355H-ATHI-O system, Oxford Lasers). For electrochemical characterization work, a 1 mm diameter disc electrode was laser-cut from a 460 μm-thick BDD wafer (Electrochemical Processing Grade). For these studies, only the growth face was exposed to solution, which had been mechanically polished to <10 nm roughness.^49^

To clean and oxygen-terminate the electrode surfaces, the laser-cut BDD electrodes were placed in boiling concentrated sulfuric acid (H_2_SO_4_; analytical reagent grade <95%, Fisher Scientific) saturated with KNO_3_ (reagent grade <99.0%, Honeywell) for 30 min, followed by placement in boiling concentrated H_2_SO_4_ for 30 min^50^ and then rinsing with deionized water. To ensure a good ohmic contact, for the larger electrode, the top 0.5 by 1 cm of the BDD rectangle was laser roughed prior to sputtering to aid adhesion. For the 1 mm disc electrode, the ohmic contact was placed on the backside of the polished cylinder. Ohmic contacts were formed via sputtering (Moorfields MiniLab 060 sputterer/evaporator) Ti/Au (10:400 nm) and then annealing at 400 °C for 5 h.^49^ For the rectangle electrode, a wire was attached to the ohmic contact with silver epoxy (Chemtronics, CircuitWorks) and the contact-wire connection was coated in non-conductive epoxy (Araldite Rapid Epoxy Adhesive, Araldite) to protect it from solution. The 1 mm diameter cylinder was sealed in a glass capillary (O.D. 2 mm; I.D. 1.16 mm, Harvard Apparatus Ltd.) using a process previously outlined.^49^

### Electrochemical Techniques

A three-electrode setup was utilized for all electrochemical experiments employing either a CHI1140B, CHI760E, or CHI1150A potentiostat (CH Instruments Inc.). The three-electrode configuration consisted of the 1 × 7 cm BDD working electrode (WE), a 1 mm disc BDD WE, a 2 mm disc Pt WE (IJ Cambria Scientific Ltd), or a 3 mm disc glassy carbon (GC) WE (IJ Cambria Scientific Ltd). As reference electrodes, a saturated calomel electrode (SCE, IJ Cambria Scientific Ltd) was used for aqueous experiments and a leak-free Ag/AgCl (LF-1.6, Alvatek) for non-aqueous experiments. A Pt coil (with an area significantly greater than the area of the 1 × 7 cm electrode) served as a counter electrode. To ensure a clean surface prior to each electrochemical characterization measurement, the disk WEs were polished using alumina paste (MicroPolish Suspension 0.05 μm, Buehler) on a polishing pad (MicroCloth PSA, Buehler), followed by polishing on an alumina free wetted polishing pad before a final rinse with deionized water.

All EC-EPR experiments were made *ex situ*, i.e., an aliquot of solution from the electrochemical cell was transferred to the EPR for analysis. The electrochemical measurements (for EC-EPR) were made using the 1 × 7 cm electrode, with a magnetic flea stirring the solution on a magnetic stirrer plate (RCT basic, IKA) to increase mass transport to the electrode. To thoroughly clean the electrode, prior to each EC-EPR measurement, the BDD electrode underwent a cathodic pre-treatment of −2.00 V vs SCE for 60 s in 0.10 M HClO_4_ (*vide infra*). For the EC-EPR experiments with ethanol, the same cathodic pre-treatment was performed but now in a solution of 5 M ethanol in 0.10 M HClO_4_. For comparison to other data presented, non-aqueous experiments performed using a leak-free Ag/AgCl reference electrode have been converted into potentials vs SCE.

### EPR Spectroscopy

EPR spectroscopy was performed on a continuous wave X-band spectrometer (Bruker EMX, Bruker) fitted with a cylindrical cavity resonator (4119HS/0207, Bruker). Aliquots of solutions from the electrochemical cell were placed in quartz EPR tubes of 1 mm inner diameter (Wilmad quartz (CFQ) EPR tubes, Sigma-Aldrich). For all measurements, the following optimized spectrometer parameters were used: a non-saturating microwave power of 10 mW; central magnetic field, 352 mT; sweep width, 10 mT; and modulation amplitude, 0.04 mT. All spectra reported are an average of 16 scans to increase the signal-to-noise ratio by roughly a factor of 4. All EPR data was fitted with simulated spin adducts using the MATLAB package EasySpin (Version 5.2.25).^51^

### Interferometry

White-light interferometry (WLM) images were collected using a 5× objective on a ContourGT profilometer (Bruker) and processed in Gwyddion (Version 2.5.2).

### Density Functional Theory (DFT) Calculations

The standard potential for DMPO oxidation was calculated from DFT simulations executed in accordance with the method used by Roth *et al*.^52^ DFT calculations were performed using the B3LYP functional, the split valence basis set 6-31 + G(d,p), and the PCM solvent continuum for solvation in water on Firefly (version 8.0.1).^53^

## Results and Discussions

**Figure 1:**
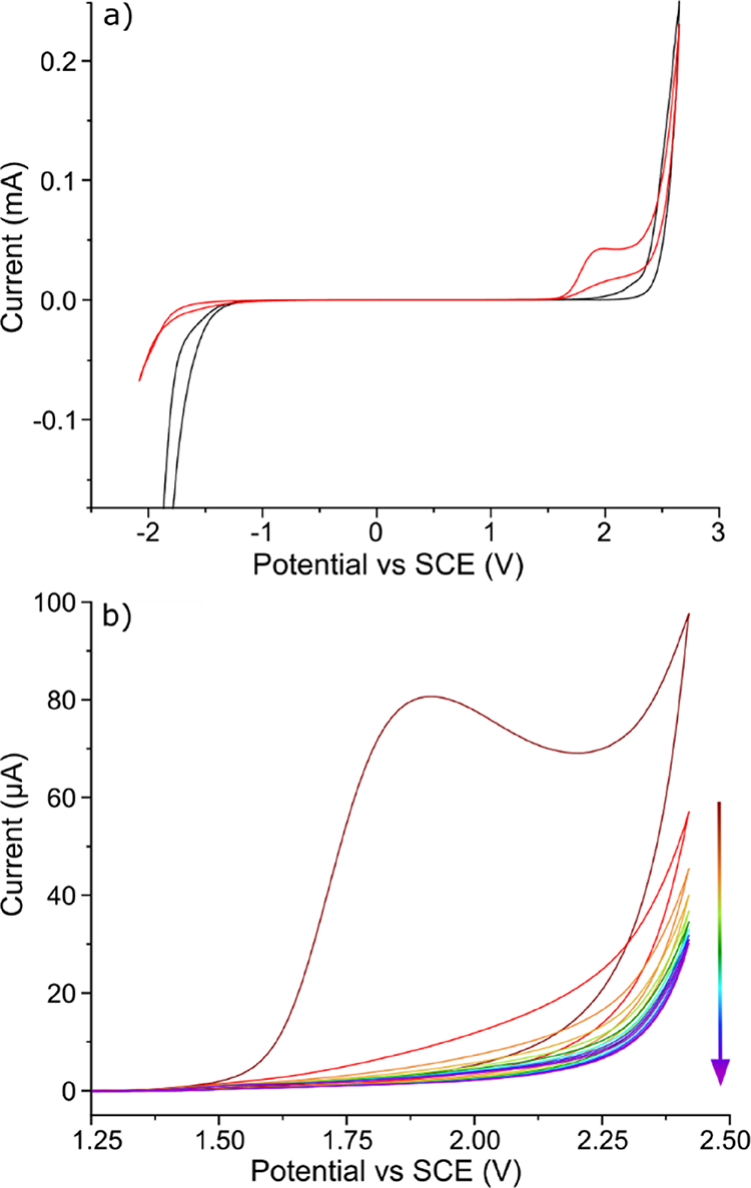
(a) CVs recording the electrochemical response of 0.10 M HClO_4_ (black line) and 10 mM DMPO in 0.10 M HClO_4_ (red line) at 0.1 V s^–1^. (b) Consecutive CVs of 30 mM DMPO in 0.30 M HClO_4_ at 0.1 V s^–1^. Arrow points to an increasing number of CVs. All data were recorded using a 1 mm diameter BDD disk electrode*.*

To explore the electrochemical characteristics of DMPO oxidation in aqueous solution, cyclic voltammograms (CVs) were recorded in a solution of 10 mM DMPO in 0.10 M HClO_4_ (red line) and 0.10 M HClO_4_ only (black line) using a 1 mm diameter disk BDD electrode at 0.1 V s^–1^, Figure 1a. Note that all scans in Figure 1 commence at 0.00 V vs SCE and then proceed first in the anodic direction up to +2.50 V vs SCE. For this work, high analyte concentrations have been chosen as these are the concentrations typically used in EC-EPR experiments to ensure excess spin trapping agents with respect to the electrochemically produced free radicals.^13,14,37^ Throughout this work, HClO_4_ was used as the supporting electrolyte due to its high stability and resilience to HO^·^ attack, as previously discussed in the literature.^54^ This is in contrast to electrolytes such as sulfuric acid (H_2_SO_4_) where the sulfate anion is susceptible to HO^·^ attack, producing sulfate (SO_4_^·^^–^) radicals and peroxodisulfate anions.^54,55^ Acidic solutions are also desirable as it is thought that low pH conditions enhance HO^·^ electrochemical production.^56^

In the anodic region in Figure 1a, for the CV recorded in the solution containing 10 mM DMPO in 0.10 M HClO_4_, an oxidative current peak (*E*_p_ = +1.90 V vs SCE) can be seen (red line). This peak is not present in the CV recorded in 0.10 M HClO_4_ only (black line). This peak in the current is attributed to the electrochemical oxidation of DMPO. Past this peak, at ca. +2.3 V vs SCE the current starts to increase rapidly due to the oxidation of water. No peak is observed on the reverse scan, until ca. −1.80 V vs SCE, where the increasing current signal is due to the electrochemical reduction of protons in solution. This data signifies that the oxidation of DMPO is not electrochemically reversible (*vide infra*) on the timescale of the voltammetric scan. On Pt and GC electrodes, DMPO shows peak electrochemical oxidation currents at ca. +1.65 V vs SCE and +1.70 V vs SCE, respectively (under the same solution and scan conditions as for Figure 1a), as shown in the Supporting Information (SI.1), Figure S1. As the oxidative peak potentials are only slightly less positive than for those seen on BDD, the data suggests that the electrode material only has a small influence on the electron transfer kinetics and mechanism of DMPO oxidation.

To examine the oxidative response in more detail, Figure 1b shows 10 consecutive CVs recorded for 30 mM DMPO in 0.30 M HClO_4_ at 0.1 V s^–1^ on a 1 mm BDD disk electrode. A clear diminution in the peak current response is seen with an increasing scan number. Even after just one scan, the current at the peak potential has dropped by nearly 90%. Such a drop in current suggests a possible blocking of the electrode surface by products of the DMPO oxidation reaction. Such behavior is commonly observed with, for example, the electrochemical oxidation of catecholamines.^57^ To explore this phenomenon further, and in particular establish whether film formation was visible on the surface, interferometric scans of the electrode surface were recorded, SI.2, Figures S2 and S3. For this experiment, two BDD electrodes were used (reflective of the two used in this study), which differed primarily in surface roughness. The two electrodes were held at two separate potentials of +1.70 V (DMPO oxidation only) and +2.50 V (DMPO and water oxidation) vs SCE for times ≥5 min in 10 mM DMPO and 0.10 M HClO_4_. Between experiments, the electrodes were cleaned by alumina polishing and rinsing. As Figures S2 and S3 show, there is evidence of film formation on the BDD electrodes, which also increases in prominence with increasing roughness of the electrode.

**Figure 2:**
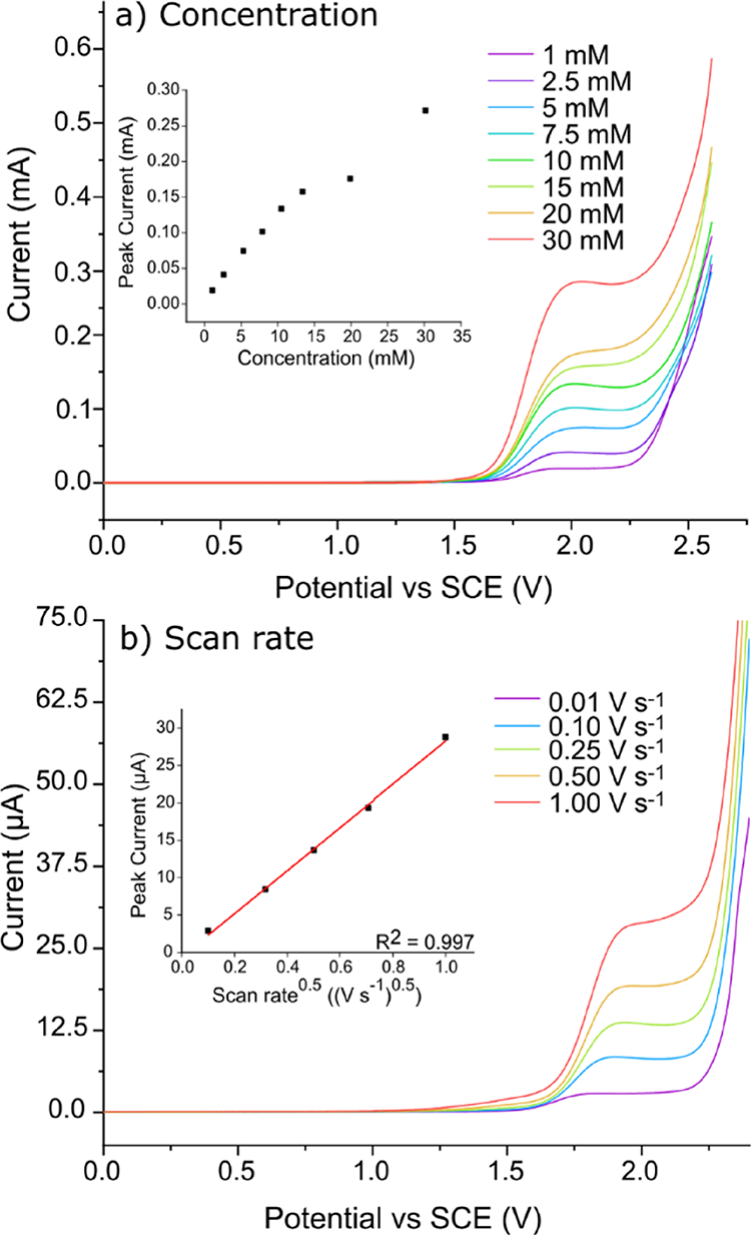
(a) LSV data for DMPO oxidation over the DMPO concentration range of 1 to 30 mM in 0.10 M HClO_4_ at 0.5 V s^–1^. The inset shows the current magnitude (recorded at *E*_p_ = +1.90 V vs SCE) with respect to the concentration over this range. (b) LSV data for 1 mM DMPO in 0.10 M HClO_4_ at different scan rates (0.01 to 1 V s^–1^). The inset shows the linearity of the peak current with respect to the square root of scan rate. All data were recorded using a 1 mm-diameter BDD disk electrode.

To explore the effect of DMPO concentration, LSVs were recorded on the 1 mm disk BDD electrode at 0.5 V s^–1^ over the DMPO concentration range of 1 to 30 mM in 0.10 M HClO_4_, as shown in Figure 2a. The higher scan rate was adopted to minimize any fouling effects over the lifetime of an individual scan. To ensure a clean electrode for each new concentration, the electrode was cleaned using alumina polishing and rinsing between solutions. To understand the relationship between the scan rate and current response, LSVs were also recorded over the scan rate range of 0.01 to 1 V s^–1^ in a solution containing 1 mM DMPO in 0.10 M HClO_4_ (Figure 2b). Here, a lower concentration was implemented to minimize fouling effects over the lifetime of an individual scan.

In Figure 2a, as the concentration of DMPO increases, the peak current increases in a linear manner over the range 1–15 mM. At the higher concentrations (>15 mM), the peak currents deviate slightly from this linear progression, most likely due to unavoidable fouling effects on the electrode surface during the scan, exacerbated by the increased concentration. The inset in Figure 2b shows that the characteristic peak current also scales linearly with the square root of scan rate, which is typically indicative of a diffusion-controlled electron transfer process.^58^

For effective use in EC-EPR, spin trapping of HO^·^ by DMPO to create the spin adduct DMPO-OH^·^ should occur only by eq 2 and not via the false positive routes shown in eqs 3 and 4. Thus, the importance of eqs 3 and 4 were investigated further. To explore the role of water as a nucleophile capable of attacking DMPO (via the Forrester–Hepburn mechanism, eq 4), 7.5 mM of DMPO was left in 0.10 M HClO_4_ and EPR spectra were recorded over a period of 40 hrs, every ca. 3 min. Over 40 h, no DMPO-OH^·^ signal was detected, indicating that water’s nucleophilic capabilities are not strong enough to result in DMPO-OH^·^ formation on the timescales appropriate for EC-EPR. Hence, in this system, complications from the Forrester–Hepburn mechanism (eq 4) can be ignored.

**Figure 3:**
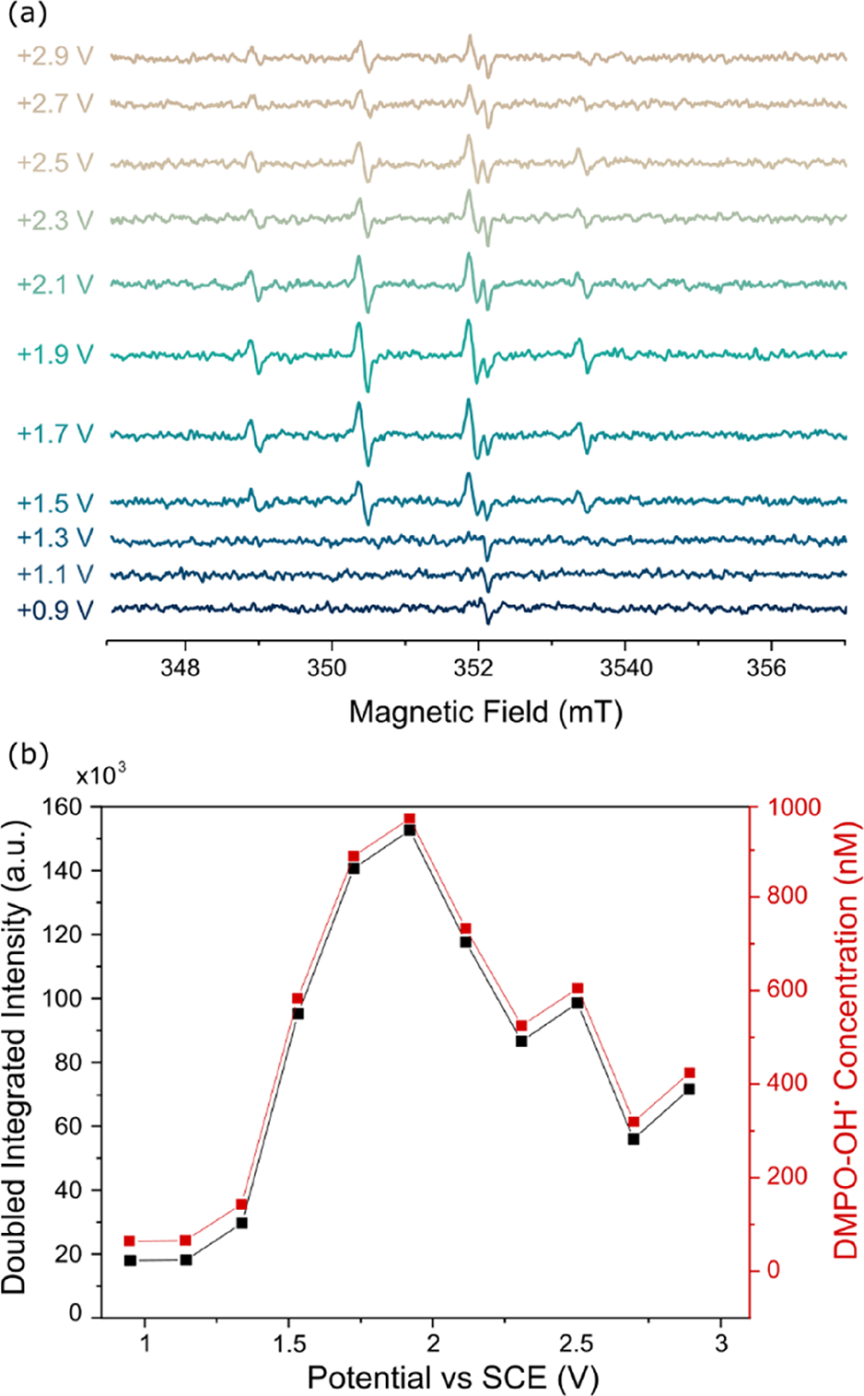
(a) EPR spectra for 5 min electrolysis of 10 mM DMPO in 0.10 M HClO_4_ at constant potentials of +0.90, +1.10, +1.30, +1.50, +1.70, +1.90, +2.10, +2.30, +2.50, +2.70, and + 2.90 V vs SCE using a 1 × 7 cm double-sided rectangle BDD electrode with an immersion depth of ca. 5 cm. (b) Plot of double integrated intensity (black) and concentration (red) of DMPO-OH^·^ extracted from EPR spectra vs the applied potential.

However, given the data in Figures 1 and 2, which show that DMPO can be electrochemically oxidized in water at potentials much lower than the thermodynamic electrode potential for HO^·^ production (*E* = +2.38 V vs SCE for pH 1.8), the impact of inverted spin trapping (eq 3) was explored further. Figure 3 shows the EPR detection of DMPO-OH^·^ as a function of applied electrode potential (vs SCE), starting from +0.90 V up to +2.90 V vs SCE, increasing in steps of 0.20 V for a generation time of 5 min. The solution contained 10 mM DMPO in 0.10 M HClO_4_, which is a typical DMPO concentration in EC-EPR detection studies of HO^·^,^13,14,37^ with the DMPO in large excess, compared to the electrogenerated radical concentration. For these measurements, the 1 × 7 cm rectangular BDD electrode was dipped into solution to an immersion depth of ca. 5 cm (with both electrode surfaces active). Such an electrode provides a large surface area, which when coupled with a suitably long generation time maximized the concentration of any product formed.

For each applied potential investigated, an aliquot of solution was removed for placement in the EPR and the spectrum was recorded. Note that for each potential, a fresh DMPO solution is made up just prior to measurement. During this 5 min timescale (and dependent on the applied potential), as Figure 1b and Figures S2 and S3 indicate, electrode fouling cannot be completely ignored (*vide infra*). To ensure a clean electrode at the start of each new applied potential experiment, this large surface area electrode was electrochemically cleaned by applying a cathodic pre-treatment (−2.00 V vs SCE for 60 s in 0.10 M HClO_4_). Pre-treatment selection for this electrode is described in SI.3. Interestingly, it is also shown in SI.3 that even just rinsing the electrode surface in distilled water will largely recover the original electrochemical response for DMPO, suggesting that any film formed is not well adhered to the surface and can be easily removed.

As seen in the EPR spectra in Figure 3a in the potential range + 0.90 to +1.30 V vs SCE, the only signal present is that attributed to the D′ signature at 352 mT,^59^ arising from the EPR quartz tube. This signal is present in all spectra though the intensity is orientation dependent, so the size of this signature varies. Starting from a potential of +1.50 V vs SCE and present at all more positive potentials, the EPR spectra in Figure 3 shows a distinct 1:2:2:1 pattern, which is characteristic of the spin adduct DMPO-OH^·^. To confirm this identification, the spectra were fitted. The extracted hyperfine coupling (A) values of *A*_N_ = 1.5 mT and *A*_H_ = 1.5 mT are in agreement with those expected for DMPO-OH^·^.^44^ Based on the thermodynamic potential for HO^·^ production at this pH (+2.38 V vs SCE) and the voltammetric range for DMPO oxidation, observation of DMPO-OH^·^ signals at potentials from +1.50 to +2.10 V vs SCE must originate from DMPO electrochemical oxidation.

It is further interesting to analyze how the DMPO-OH^·^ concentration varies as a function of applied potential, as shown in Figure 3b. The concentration of DMPO-OH^·^ has been determined by fitting of the four peaks due to DMPO-OH^·^ and then doubly integrating to estimate the area under the peaks. This double integrated intensity is translated into a concentration of DMPO-OH^·^ using a 4-hydroxy TEMPO calibration, SI.4. As Figure 3b shows, the DMPO-OH^·^ concentration rises as the potential increases from +1.30 to +1.90 V vs SCE and then overall falls with small intermittent rises over the potential range of +2.10 to +2.90 V vs SCE.

**Scheme 1:**
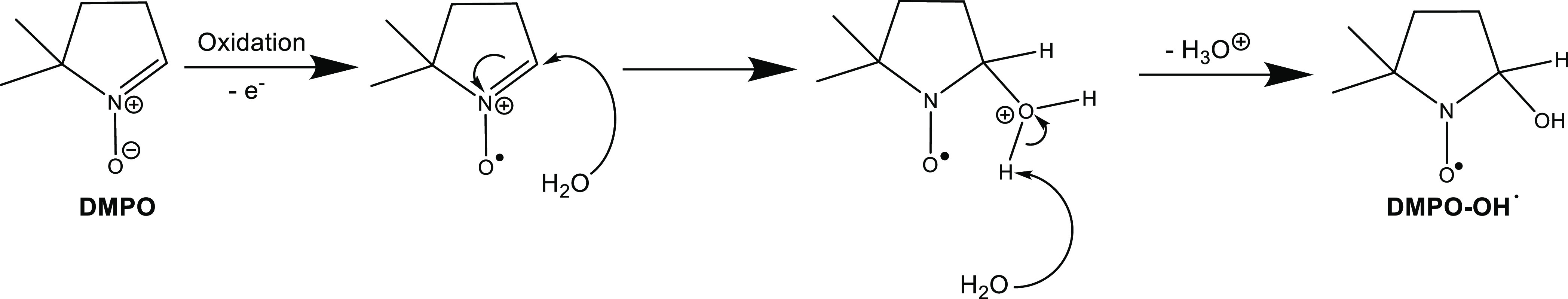
Schematic for Proposed Mechanism for the Electrochemical Oxidation of DMPO and Subsequent Attack of Water

Given the EC-EPR data in Figure 3 and observation of a DMPO-OH^·^ signal in the potential region associated with DMPO electrochemical oxidation, we postulate the following mechanism for DMPO-OH^·^ formation, Scheme 1. Many organic compounds undergo chemically irreversible electron transfer, forming a highly reactive charged or radical species with a short half-life.^60^Scheme 1 describes one electron oxidation of DMPO to form the reactive radical, DMPO^+^^·^ - an electron transfer step (E) - followed by subsequent rapid attack of the nucleophile water and H_3_O^+^ loss - a solution chemical reaction, C_sol_ - *i.e.*, an EC_sol_ process. The number of electrons transfer (*n*) during the electrochemical oxidation step must be odd due to the generation of a paramagnetic species (Figure 3). One electron transferred is the most feasible. This mechanism also fits with the observed lack of a reverse wave for DMPO oxidation in Figure 1, which highlights the instability of the initial radical species produced on the timescale of the electrochemical voltammogram. Pei and co-workers^45^ claim that DMPO-OH^·^ formation via the inverted spin trapping route (eq 3) requires attack of OH^–^ and thus is not possible in acidic solutions. Their argument was based on the lack of reactivity of OH^–^ (and water) with DMPO rather than consideration of the reaction of OH^–^ (and water) with the electrochemically produced DMPO^+·^. No EPR data in the potential region for DMPO oxidation was presented to support their claim. We suggest that as DMPO^+·^ is a significantly better electrophile than DMPO; it will therefore react, like many other related oxonium or iminium ions, with water (under acidic conditions), as supported by our experimental data.

If we assume Figures 1 and 2 are reflective of an EC_sol_ process, it is useful to consider the magnitude of the observed currents. DigiElch was used to model the EC_sol_ process, assuming the following: (i) *n* = 1 for the electron transfer process; (ii) a diffusion coefficient for DMPO of 9.9 × 10^–6^ cm^2^ s^–1^, estimated using the Wilke–Chang model (detailed in SI.5);^61^ (iii) 10 mM DMPO; (iv) 0.1 V s^–1^ scan rate; (v) a standard potential of +1.70 V vs SCE for DMPO/DMPO^+^^·^ (taken from DFT calculations); (vi) a transfer coefficient = 0.5; (vii) temperature = 298.15 K; and (vi) a 1 mm diameter disk electrode. When accounting for the chemical step and using a significantly high value for an effective solution rate constant (*k*_sol_ ≫ 10 s^–1^) such that a reverse peak is no longer seen, DigiElch predicts a peak current of 23.0 μA (Figure S6, SI.6), which is slightly larger and shifted more negative compared to the reversible electron transfer only case (Figure S6).^60^

Interestingly, experimentally, a higher peak current of 42.7 μA is observed (Figure 1) under these experimental conditions. Thus, we speculate that once DMPO has been oxidized to DMPO^+·^, which through reaction with water converts to DMPO-OH^·^ (Scheme 1), the potential is such that the DMPO-OH^·^ can easily undergo further electron loss, i.e*.*, an EC_sol_E mechanism. Possible electron loss pathways for DMPO-OH^·^ and the resulting oxidation products (HDMPO and HDMPN) are shown in SI.7, Figure S7. DFT data suggests that the oxidation of DMPO-OH^·^ and HDMPO is more favorable than DMPO.^45^ Hence, for the currents passed in this potential range, the effective number of electrons transferred is greater than one, due to more than one process occurring. This leads to a current magnitude greater than that predicted based on EC_sol_ only.

**Figure 4:**
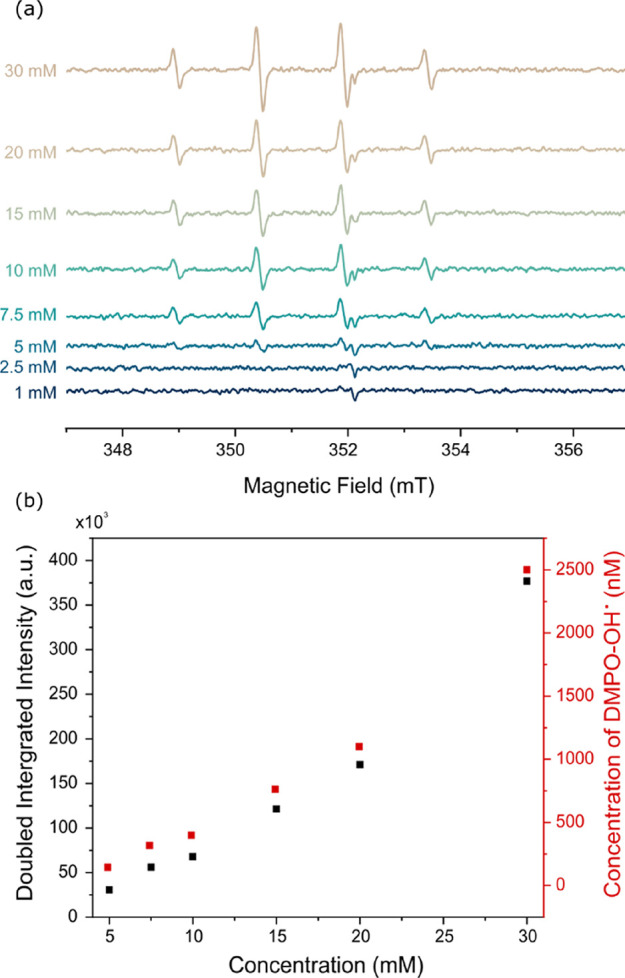
(a) EPR spectra for 5 min electrolysis of 1, 2.5, 5, 7.5, 10, 15, 20, and 30 mM DMPO in 0.10 M HClO_4_ at a constant potential of +1.90 V vs SCE using a 7 by 1 cm double-sided BDD electrode with an immersion depth of ca. 5 cm. (b) Plot of double integrated intensity (black) and concentration (red) of DMPO-OH^·^ extracted from (a).

To further verify that the DMPO-OH^·^ signals below +2.38 V vs SCE were due to the electrochemical oxidation of DMPO followed by water nucleophilic attack, EPR measurements were carried out by varying the concentration of DMPO from 1 to 30 mM in 0.10 M HClO_4_, Figure 4. Prior to EPR analysis, the DMPO was electrochemically oxidized by holding the potential at +1.90 V vs SCE for 5 min. As can be seen in Figure 4a, the characteristic DMPO-OH^·^ (1:2:2:1) splitting pattern becomes evident for DMPO concentrations of ≥5 mM, with the signal intensity growing in magnitude as the concentration of DMPO increases. Note that the signal for DMPO-OH^·^ obtained in 5 mM DMPO (at this potential and time) is on the threshold of the limit of detection. Figure 4b shows a plot of the intensities and resulting DMPO-OH^·^ concentrations, with the DMPO-OH^·^ concentration increasing with increasing DMPO concentration, up to ca. 2.5 μM for a 30 mM DMPO concentration. This data further supports the hypothesis that electrochemical oxidation of DMPO is the source of the DMPO-OH^·^ species at this potential.

Given the data presented in Figures 3 and 4, there are two issues to consider further. The first is why does the EPR signal for DMPO-OH^·^ have the potential dependent shape as shown in Figure 3? For a DMPO concentration of 10 mM, the concentration of DMPO-OH^·^ rises to a maximum of ca. 0.9 μM at +1.90 V vs SCE and then falls by almost a half when moving more positive to +2.30 V vs SCE. The concentration drops further to ca. 0.4 μM at the highest potential investigated (+2.90 V vs SCE). To explore what happens when the potential was increased even further, SI.8, Figure S8 shows data points (in red) for EPR detection of DMPO-OH^·^ as a function of applied electrode potential, starting from +3.00 V vs SCE in steps of 0.5 V up to +5.00 V vs SCE. The signal for DMPO-OH^·^ continues to fall further as the potential is increased to +5.00 V vs SCE.

The concentration of DMPO-OH^·^ in the region of DMPO oxidation appears to mirror the voltammetric wave shape for DMPO oxidation. However, it is surprising that as the potential moves into a region where additional DMPO-OH^·^ formation from the oxidation of water is expected, the concentration of DMPO-OH^·^ instead falls. Possible reasons for this could be that as the potential increases, the overpotential for electrochemical oxidation pathways (as shown in SI.7), which remove DMPO-OH^·^ from the solution, will also increase. Although, we note that no other paramagnetic products (e.g., DMPO-X^·^ and HDMPO-OH^·^) were detected even at these higher potentials in Figure S8. Bubble formation, which is more prominent at the higher potentials, will also reduce the active electrode surface area and amount of product (for the time period considered). It is also possible that film formation (Figures S2 and S3) is exacerbated at higher potentials, which will also act to block the electrode surface.

The second issue is, for electrode potentials where production of HO^·^ is possible from the oxidation of water, how much of the DMPO-OH^·^ EPR signal is from direct water oxidation (eq 1) and how much is from electrochemical oxidation of DMPO (Scheme 1 and eq 3)? Can we ever truly quantify HO^·^ concentration via the DMPO-OH^·^ signal under conditions where the spin trap has been electrochemically oxidized? It has been discussed in the literature that the presence of an organic radical scavenger, such as ethanol, aids the differentiation of the spin adducts generated from inverted spin trapping (eq 3) to those generated from radical trapping (eq 2). This provides a possible route to differentiating between spin adducts generated from the electrochemical oxidation of DMPO from those produced via the spin trapping of HO^·^, in potential regions where both processes are thought to occur.

In theory, for the specific case of DMPO (spin trap) and ethanol (radical scavenger), once DMPO has been electrochemically oxidized, ethanol can act as a nucleophile and react through the more nucleophilic oxygen to form the DMPO-OCH_2_CH_3_ spin adduct. In contrast, when HO^·^ is electrochemically generated (eq 1), HO^·^ will react with ethanol by scavenging an α-hydrogen to generate a carbon-centered radical,^30^ which can react with DMPO to form a different DMPO-CH(OH)CH_3_^·^ spin adduct. Each ethanol-based spin adduct has distinctive splitting patterns in EPR spectroscopy enabling the two different reaction pathways to be distinguished.

**Figure 5:**
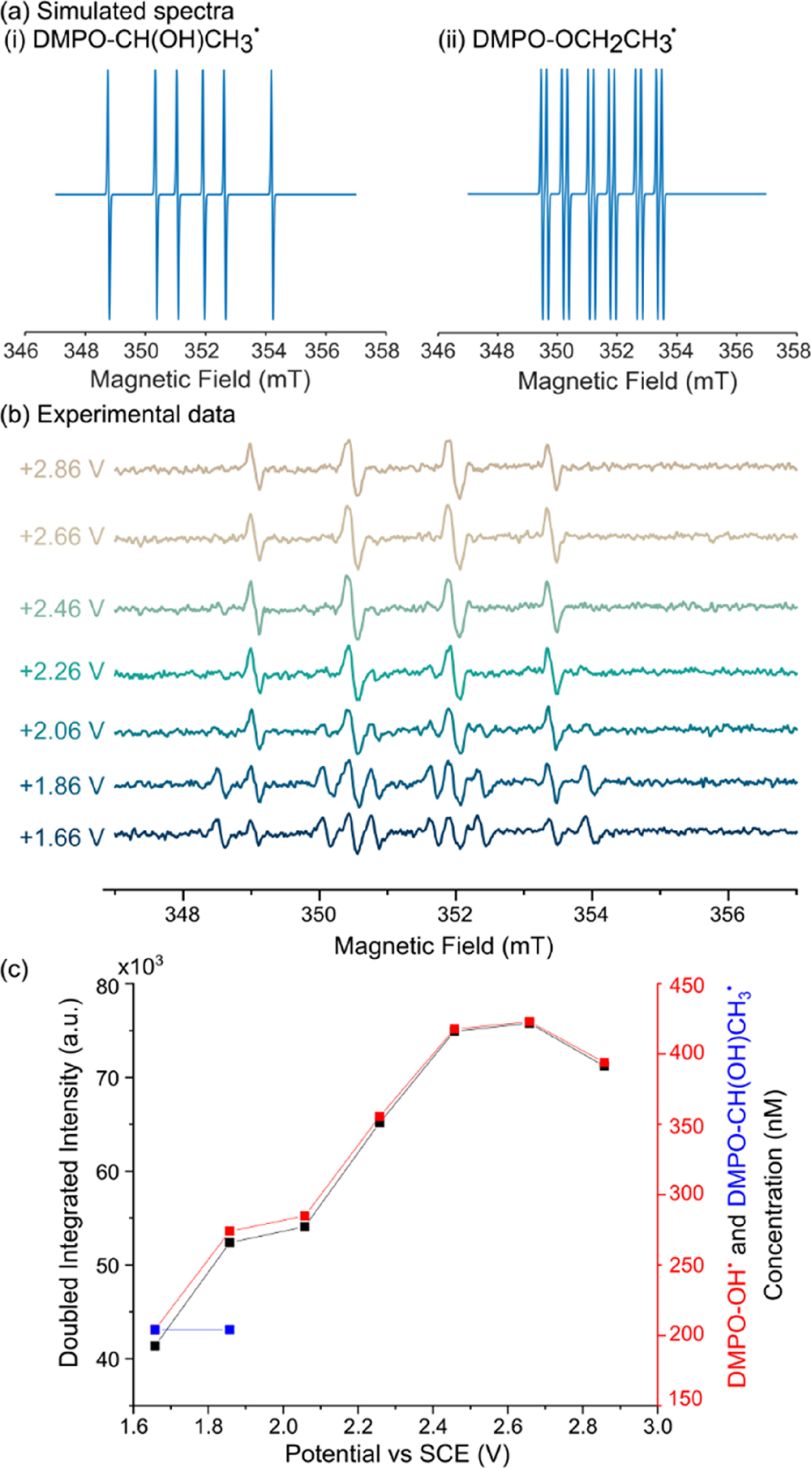
Simulated spectra of (a) (i) DMPO-CH(OH)CH_3_^·^ hyperfine coupling: *A*_N_ = 1.58 mT and *A*_H_ = 2.29 mT and (a) (ii) DMPO-OCH_2_CH_3_^·^ hyperfine coupling: *A*_N_ = 1.32 mT, *A*_H_ = 0.70 mT, and *A*_H_ = 0.19 mT.^44^ (b) EPR spectra for 5 min electrolysis of 10 mM DMPO and 5 M ethanol in 0.10 M HClO_4_ at constant potentials of +1.66, +1.86, +2.06, +2.26, +2.46, +2.66, and + 2.86 V vs SCE using a double-sided 1 × 7 cm rectangle BDD electrode with an immersion depth of ca. 5 cm. (c) Plot of double integrated intensity (black) and concentrations of DMPO-OH^·^ (red) and DMPO-CH(OH)CH_3_^·^ (blue) extracted from EPR spectra vs the applied potential.

Figure 5a presents the simulated spectra for (i) DMPO-CH(OH)CH_3_ (HO^·^ route) and (ii) DMPO-OCH_2_CH_3_^·^ (DMPO oxidation route). Figure 5b shows EPR spectra recorded after 5 min electrolysis of 10 mM DMPO and 5 M ethanol (0.083 mole fraction) in 0.10 M HClO_4_ at potentials between +1.66 to +2.86 V vs SCE in 0.20 V increment steps. This covers a region where only oxidation of DMPO occurs, to potentials where HO^·^ will also be electrochemically produced from water oxidation. A sufficiently high concentration of ethanol is required for this experiment so that ethanol can act as a competing nucleophile with water for the oxidized DMPO (DMPO^+·^). To clean the electrode prior to each measurement, the cathodic pretreatment in 0.10 M HClO_4_ and 5 M ethanol was employed.

The spectrum of DMPO-OH^·^, which has four peaks in a 1:2:2:1 ratio, is presented in Figures 3 and 4. The spectrum for DMPO-CH(OH)CH_3_^·^ has six peaks in equal intensities with hyperfine couplings of *A*_N_ = 1.58 mT and *A*_H_ = 2.29 mT, while the spectrum for DMPO-OCH_2_CH_3_^·^ has 12 peaks in equal intensity with hyperfine couplings of *A*_N_ = 1.32 mT, *A*_H_ = 0.70 mT, and *A*_H_ = 0.19 mT. In the experimental data in Figure 5b, a 10-peak signature can be clearly observed at both +1.66 and +1.86 V vs SCE. Note that for ease of data display, different intensity scales have been used so that all peaks can be clearly seen. The actual intensities are given in Figure 5c. Fitting of these spectra indicates that the 10-peak signal is due to both DMPO-CH(OH)CH_3_ and DMPO-OH^·^.

**Scheme 2:**
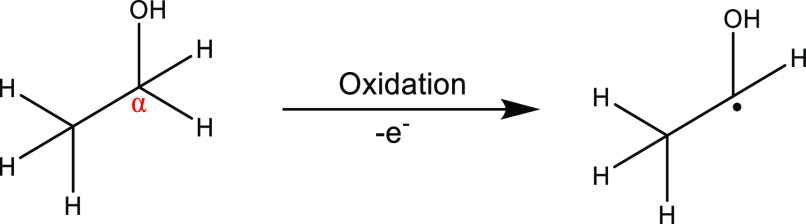
Electrochemical Oxidation of Ethanol at the α-Hydrogen

The presence of DMPO-OH^·^ is expected at these lower potentials, as electrochemical oxidation of DMPO can occur in this acid (0.917 mole fraction)–ethanol solution. However, it is surprising to see DMPO-CH(OH)CH_3_^·^, as at these potentials, no electrochemically generated HO^·^ radicals from water oxidation should be present to oxidize the ethanol. To explain the presence of DMPO-CH(OH)CH_3_^·^, we thus explored whether electrochemical oxidation of ethanol was playing a role in formation of the radical adduct. As shown in SI.9, Figure S9, in ethanol, with 0.10 M TBAB added to increase solution conductivity, the onset potential for ethanol oxidation is ca. +1.6 V vs SCE, at 0.1 V s^–1^, on a 1 mm diameter disk BDD electrode. This data demonstrates that ethanol electrochemical oxidation occurs at less positive potentials than water oxidation (in 0.1 M HClO_4_) on a BDD electrode (see Figure 1a) and is thus electrochemically more facile. Electro-oxidation of ethanol can result in the extraction of α-hydrogen producing the carbon-centered radical, ^·^CH(OH)CH_3_, as shown in Scheme 2, which then reacts with DMPO, resulting in the observed DMPO-CH(OH)CH_3_^·^ signals. The lower onset potential for ethanol oxidation explains the presence of the DMPO-CH(OH)CH_3_^·^, which is most easily observed at the lower applied potentials.

Electrochemical oxidation of ethanol would thus be expected to hinder differentiation of DMPO-OH^·^ from inverted spin trapping (eq 3) vs direct electrochemical production of HO^·^ (eq 2). Interestingly, Figure 5b shows that as the potential increases, the product distribution between DMPO-OH^·^ and DMPO-CH(OH)CH_3_ moves in favor of DMPO-OH^·^, resulting in DMPO-OH^·^ becoming the only species observable in the EPR spectrum, at potentials of ≥+2.46 V vs SCE. Figure 5c shows the concentrations of DMPO-OH^·^ and DMPO-CH(OH)CH_3_^·^. While DMPO-CH(OH)CH_3_^·^ concentrations could be extracted from the data at 2.06 and 2.26 V, as the signal to noise ratio has considerably reduced, there would be significantly more error on the values obtained. These concentrations have thus been omitted from Figure 5c. It is suspected that a small amount of DMPO-CH(OH)CH_3_^·^ is still present at the even higher potentials but is now overwhelmed by the much larger DMPO-OH^·^ signal. This change in product distribution and the observation of only DMPO-OH^·^ at potentials of ≥+2.46 V vs SCE suggest the presence of a second mechanism facilitating DMPO-OH^·^ formation. We attribute this to the electrochemical generation of HO^·^ radicals from water oxidation on BDD (water is greatly in excess compared to ethanol).

It is also noted in Figure 5 that no evidence of the oxygen-centered ethoxy radical (CH_3_CH_2_O^·^) is observed at potentials where it is believed that the HO^·^ can oxidize ethanol to produce ethoxy radicals. This could be due to the low mole fraction (0.083) of ethanol in the mixture or DMPO-OCH_2_CH_3_^·^ undergoing a further one electron oxidation to acetaldehyde, an EPR-silent nitrone.^34^ In this study, again no evidence for further electrochemical oxidation of DMPO-OH^·^ to the paramagnetic species DMPO-X^·^ and/or HDMPO-OH^·^ (SI.7) was observed, even up to +5.00 V vs SCE (SI.8). We note that Pei and co-workers observed a triplet signal (unattributed) in addition to DMPO-OH^·^ at +5.62 V vs SHE on a titanium suboxide electrode.^45^

**Figure 6:**
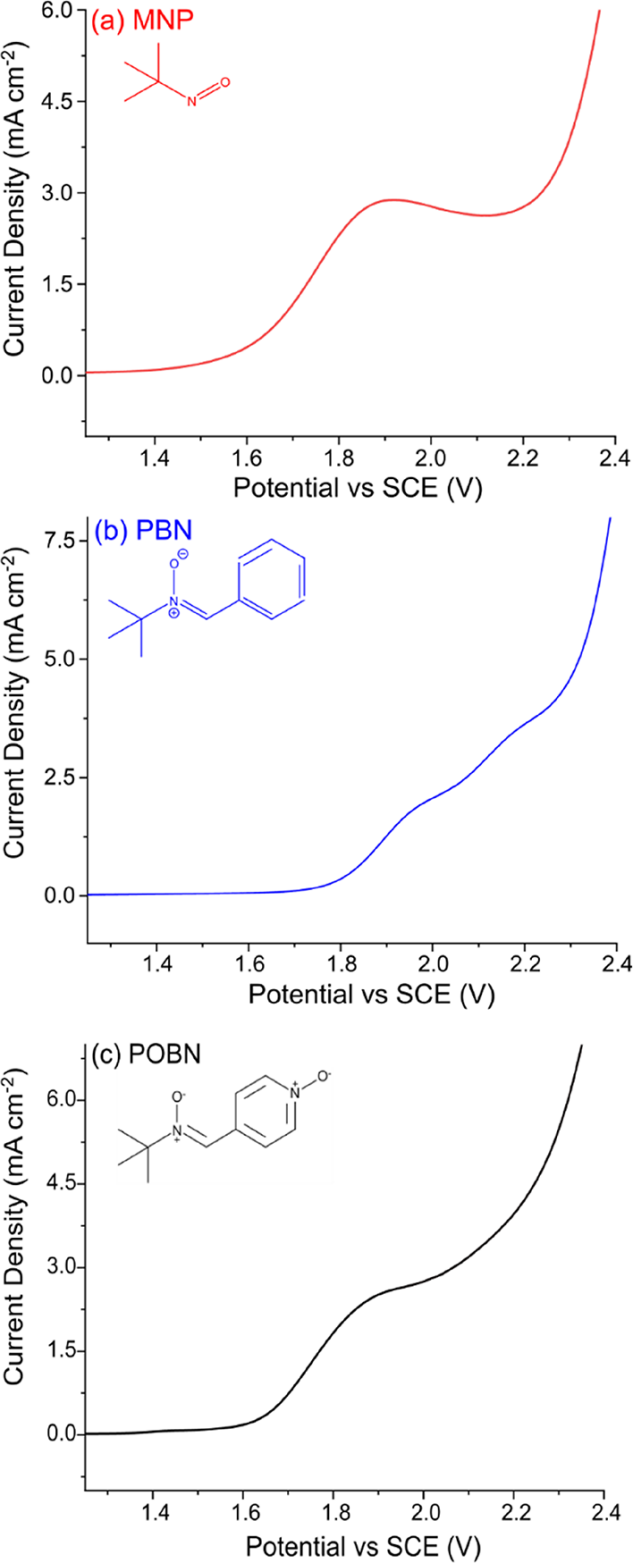
Oxidative windows for 10 mM (a) MNP dimer, (b) PBN, and (c) POBN in 0.10 M HClO_4_ at a scan rate of 0.1 V s^–1^ on a 1 mm BDD electrode.

Although, DMPO is the most ubiquitously used spin trap for EC-EPR, it is expected that other commonly used spin traps will be prone to the same pitfalls experienced by DMPO, when the potential required to electrochemically generate the radical is greater than the oxidation potential of the spin trap. To date, voltammetric characterization measurements have been made on PBN and other spin traps using a Pt electrode.^42^ However, in most cases, the water oxidation currents obscured any possible signal due to oxidation of the spin traps investigated and oxidation potentials had to be inferred by using non-aqueous solutions.^42^ By using a BDD electrode, as water oxidation is significantly electrocatalytically retarded, it should be possible to observe the oxidation signals of many different spin traps in aqueous solutions. This is illustrated by the data shown in Figure 6**,** which presents the electrochemical oxidative window for three common spin traps (PBN and the previously un-investigated MNP dimer and POBN) all at 10 mM in 0.10 M HClO_4_ recorded at 0.1 V s^–1^ on a 1 mm-diameter disk BDD electrode. MNP contains a nitroso functional group to stabilize the radical, while PBN and POBN contain nitrone functional groups.

Similar to DMPO, a clear peak in the anodic region is observed at +1.91 and + 1.88 V vs SCE for MNP dimer and POBN, respectively, in Figure 6. For PBN, two oxidation peaks can be observed at +1.97 and + 2.19 V vs SCE. Given the structural similarity of the spin traps, it is perhaps not surprising that they electrochemically oxidize at similar potentials. The observation of oxidation peaks for these three spin traps, before water oxidation on BDD, highlights the importance of accounting for electrochemical oxidation of the spin traps themselves when interpreting EC-EPR data. Possible mechanisms for the electrochemical oxidation of these molecules are postulated in SI.10.

## Conclusions

This study has demonstrated that the use of EPR, in combination with spin trap labels, to detect HO^·^ generated electrochemically from water oxidation is challenging. This is primarily due to the spin trap (here we use DMPO) undergoing electrochemical oxidation at potentials less positive than that of water, resulting in the same spin trap adduct (DMPO-OH^·^) as would be produced from OH^·^–DMPO interactions. For DMPO, in acidic aqueous media, using a BDD electrode, electrochemical oxidation of DMPO commenced at +1.40 V vs SCE, reaching a peak current at +1.90 V vs SCE. The current due to water oxidation was observed to start rising rapidly at the more positive potential of ca. +2.3 V vs SCE. EC-EPR measurements made in the DMPO oxidation potential region confirmed the formation of the spin adduct DMPO-OH^·^. This was postulated to arise from the one electron oxidation of DMPO to DMPO^+^^·^ and subsequent reaction of DMPO^+^ with water to form DMPO-OH^·^.

When measuring the DMPO-OH^·^ concentration via EC-EPR as a function of applied electrode potential, the highest concentrations were surprisingly recorded in the region of DMPO oxidation (+1.90 V vs SCE), which decreased as the potential was increased into the water oxidation region. Such behavior was attributed to the removal of DMPO-OH^·^ from solution via subsequent electrochemical oxidation and the formation of fouling products (films) and bubbles on the electrode surface. The data suggested that quantification of the true concentration of OH^·^ generated from water oxidation via EC-EPR is problematic. The use of different cell geometries, such as flow cells and/or rotating disk electrodes,^63^ could be beneficial to minimize fouling events and prevent further oxidation of DMPO-OH^·^, as the product is swept away from the electrode surface. Further investigations are required. This study also demonstrated that adding a radical scavenger, in this case ethanol (5 M), to confirm the presence of HO^·^ from water oxidation, via EPR, also has its challenges for very similar reasons as above, i.e*.,* the radical scavenger will also get electrochemically oxidized at potentials less positive than that of water oxidation.

Finally, the understanding gained in this paper applies not only to DMPO but also any molecule being used to spin trap HO^·^ electrochemically generated from the oxidation of water. Here, we investigated three spin trap systems, MNP dimer, PBN, and POBN using a BDD electrode, and all three showed an oxidative response before the onset of water oxidation. Conversely, the understanding also applies beyond electrochemically produced HO^·^ and to the use of DMPO for the detection of other electrochemically generated free radicals, for example, the generation of chlorine (Cl^·^) and SO_4_^·–^ free radicals from the electrochemical oxidation of Cl^–^ and SO_4_^2–^ (*E*^0^ = +2.19 V vs SCE^10^). For both of these ions, DMPO oxidation will also be problematic, resulting in false positive spin adducts due to attack of the oxidized DMPO^+·^ by Cl^–^ or SO_4_^2–^, both of which are stronger nucleophiles than water.^62^

## Supporting Information Available

DMPO electrochemical behavior on GC and Pt; fouling data; pre-treatment data; 4-hydroxy TEMPO calibration; estimation of *D*; digital simulations; oxidation pathways of DMPO; EC-EPR up to 5 V; CV of ethanol; electrochemical oxidation of other spin traps (PDF)
