# Supplementary material for: Electron Paramagnetic Resonance for the Detection of Electrochemically Generated Hydroxyl Radicals: Issues Associated with Electrochemical Oxidation of the Spin Trap
Source: ACS Meas Sci Au. 2022 Sep 26;3(1):21–31. doi: 10.1021/acsmeasuresciau.2c00049 (PMC9936800; doi:10.1021/acsmeasuresciau.2c00049)
Supplement: Supplementary file 1 — tg2c00049_si_001.pdf [file tg2c00049_si_001.pdf]

Supporting Information

Electron Paramagnetic Resonance for the Detection of Electrochemically Generated Hydroxyl Radicals: Issues Associated with Electrochemical Oxidation of the Spin Trap

Emily Braxton,[1,2] David J. Fox,[1] Ben G. Breeze,[3] Joshua J. Tully,[1] Katherine J. Levey,[1,4] Mark E. Newton[3] and Julie V. Macpherson*[1]

[1] Department of Chemistry, University of Warwick, Coventry, United Kingdom

[2] Molecular Analytical Science Centre for Doctoral Training, University of Warwick, Coventry, United Kingdom

[3] Department of Physics, University of Warwick, Coventry, United Kingdom

[4] Centre for Doctoral Training in Diamond Science and Technology, University of Warwick, Coventry, United Kingdom

Contents

SI.1 DMPO Electrochemical Behavior on Platinum and Glassy Carbon

SI.2 Investigate of Fouling Film Formed by DMPO Oxidation

SI.3 Investigation of Cleaning of Oxidized DMPO Film

SI.4 Radical Concentration Calibration using 4-hydroxy TEMPO

SI.5 Estimation of Diffusion Coefficient for DMPO

SI.6 Digital Simulations

SI.7 Electrochemical Oxidation Pathways of DMPO

SI.8 Electrochemical-EPR Experiments at Higher Potentials.

SI.9 Solvent Window of 0.1 M TBAB in Ethanol

SI.10 Mechanism for Electrochemical Oxidation of MNP, PBN and POBN

SI.1 DMPO Electrochemical Behavior on Platinum and Glassy Carbon

To determine the electrochemical characteristics of 5,5-dimethyl-1-pyrroline N-oxide (DMPO) oxidation on different electrode materials, cyclic voltammograms (CVs) were collected in 10 mM DMPO in 0.10 M $HClO_4$ on a 2 mm diameter Pt electrode and a 3 mm diameter glassy carbon (GC) electrode at 0.1 V s^{-1}, shown in **Figure S1**. Note, all scans in Figure S1 commence at 0.00 V vs SCE and then proceed first in the anodic direction.

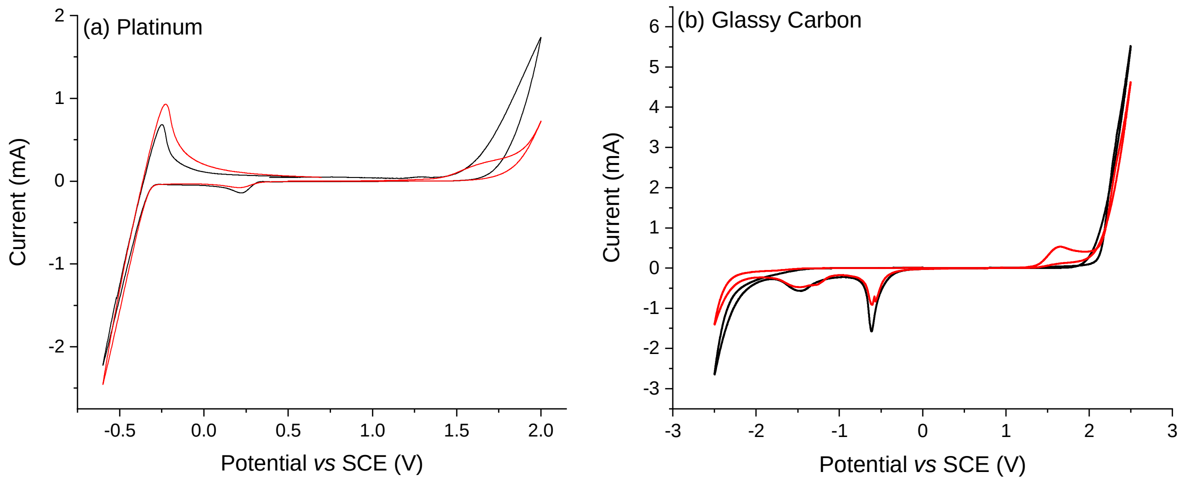

Figure S1: CVs for the electrochemical response of 0.10 M $HClO_4$ (black) and 10 mM DMPO in 0.10 M $HClO_4$ (red) on (a) 2 mm Pt disk and (b) 3 mm disk GC electrode at 0.1 V s^{-1}.

The DMPO oxidative signatures can be seen on both Pt and GC electrodes (red lines), at +1.65 V vs SCE and +1.70 V vs SCE respectively. The oxidative potentials are slightly less positive than BDD, suggesting that electrode material has a small influence on the electron transfer kinetics of DMPO oxidation.

SI.2 Investigate of Fouling Film Formed by DMPO Oxidation

To investigate whether a film on the BDD electrode formed as a result of electrochemical oxidation of DMPO, interferometry was employed in a solution containing 10 mM DMPO in 0.10 M $HClO_4$ at +1.70 and +2.50 V vs SCE. After electrolysis had completed, the electrode was removed from solution and left to air dry and then the surface morphology assessed using white light interferometry (WLI). For each potential at least five areas of the electrode surface were imaged. Note whilst we assume that removing the BDD electrode from the solution does not impact detrimentally on any film formed on the surface, we cannot be certain. The film formation on two different electrodes was studied, the first electrode was 1 × 1.5 cm in size (Electrochemical Processing Grade) and had only the polished face (~50 nm RMS) exposed to solution. WLI of this electrode is shown in **Figure S2**. The electrode potential was applied for 5 min.

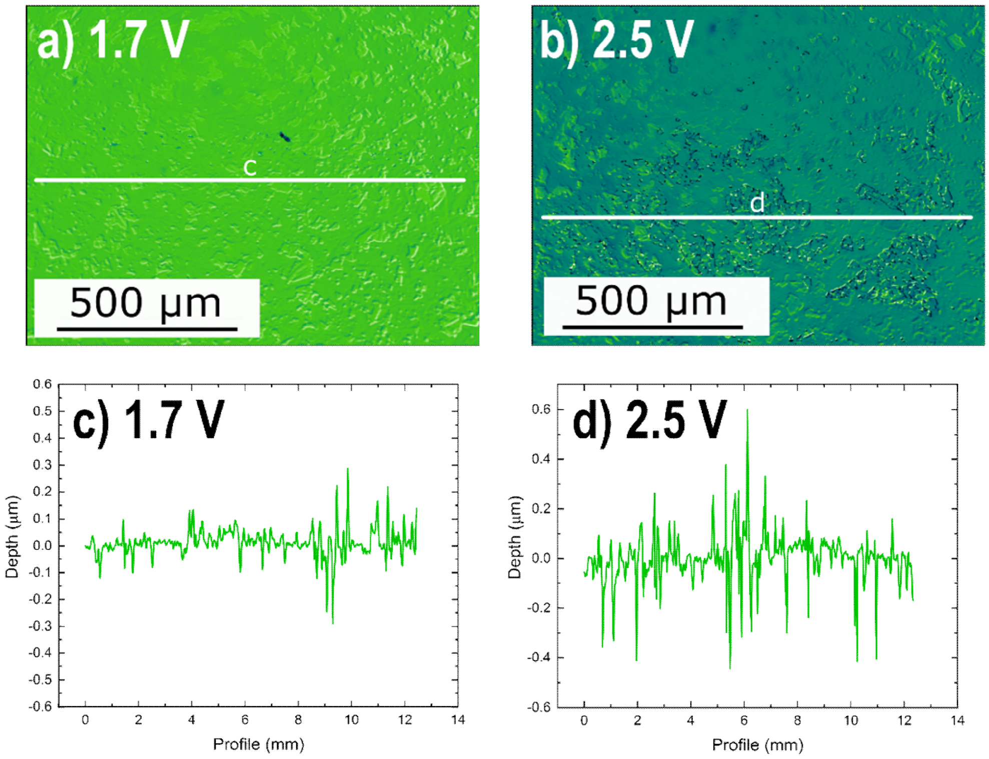

Figure S2: WLI of the surface of the 1 × 1.5 cm mechanically polished BDD electrode after a) 5 min at +1.70 V vs SCE. b) 5 min at +2.50 V vs SCE. c) Line profile across the +1.70 V vs SCE deposition image. d) Line profile across the +2.50 V vs SCE deposition image.

At +1.70 V vs SCE no clear evidence of film formation was observed. Once the deposition potential was increased to +2.50 V vs SCE, a patchy film with a honeycomb like structure and a thickness between 100 and 600 nm was observed, which covered ca. 10% of the surface.

The experiment was also performed on the larger (1 × 7 cm) BDD electrode used for EC-EPR which had both the rough (~ 10 µm RMS) growth face and smoother nucleation face (~150 nm RMS) exposed to solution. Note, it was not possible to characterize the growth face by WLI due to the surface being too rough. For each potential at least five areas of the electrode surface were imaged and a film was observed in all images. The electrode potential was applied for 10 min.

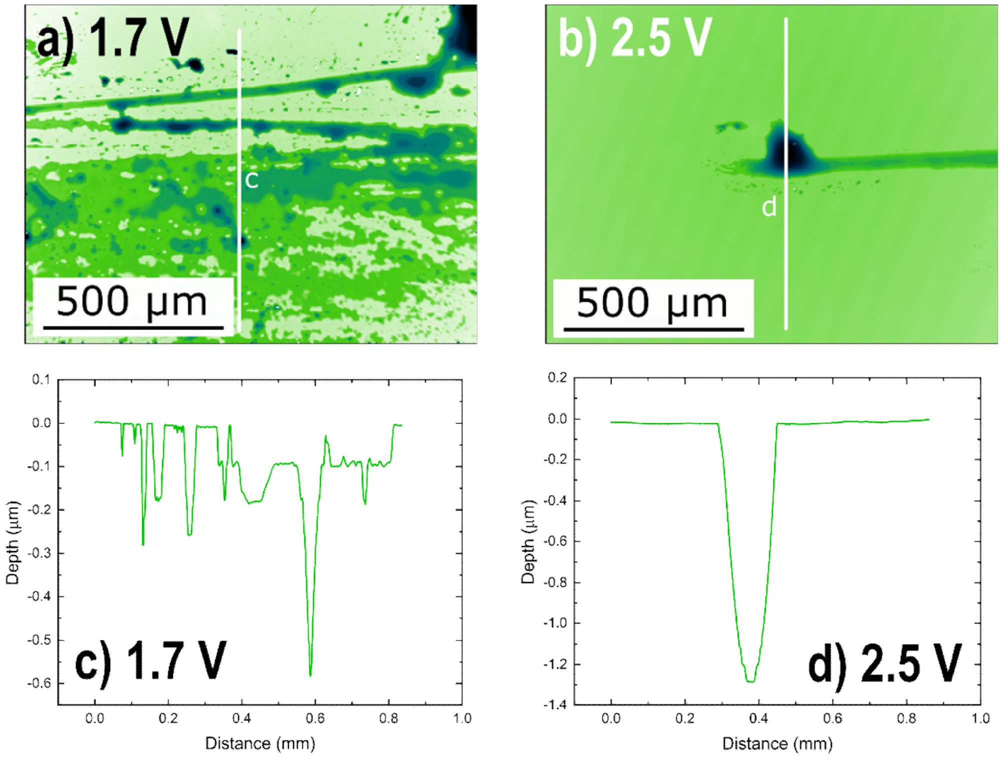

Figure S3: WLI of the surface of the 1 × 7 cm BDD electrode (nucleation face) after a) 10 min at +1.70 V vs SCE followed by scratching to remove any film formed. b) 10 min at +2.50 V vs SCE followed by scratching to remove any film formed. c,d) Corresponding line profiles across the WLI image in a) and b) respectively.

For both deposition potentials, a more prominent film was observed on this BDD surface (nucleation face). The surface was scratched with metal tweezers (BDD is scratch resistant) to provide more information on the film thickness. For the +1.70 V vs SCE film, the film thickness was found to vary between 0.10 and 1.50 µm across the electrode surface (only one image presented). For the +2.50 V vs SCE film, the film variation thickness was similar, measured as being between 0.10 and 1.25 µm thick at various sites across the surface.

SI.3 Investigation of Cleaning of Oxidised DMPO Film

To clean the electrode of any possible fouling products as indicated in **Figures 1b**, **S2** and **S3**, four different cleaning methods were investigated: (a) rinsing with deionized (distilled) water, (b) alumina polishing, (c) an applied cathodic potential (-2.00 V vs SCE for 60 s) and (d) an applied anodic treatment (+2.00 V vs SCE for 60 s). **Figure S4** shows CVs recorded in 10 mM DMPO in 0.10 M $HClO_4$ on a double sided 7 by 1 cm BDD electrode at 0.1 V s^{-1} before electrolysis (black) and after a 5 min electrolysis at +1.90 V vs SCE (red) and after one of the four cleaning steps (blue). Variations in the shapes of the DMPO CVs are attributed to the different cleaning treatments changing the BDD surface chemistry.[1,2] The in-situ electrochemical cleans were deemed most effective and less labor intensive. For these studies the cathodic clean was selected.

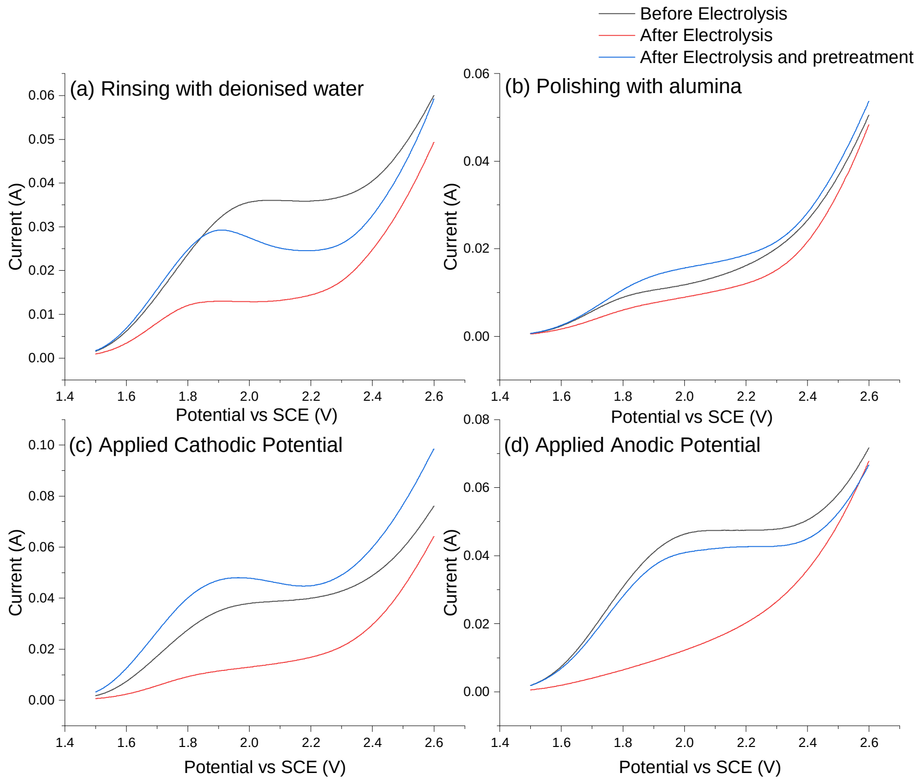

Figure S4: CVs of 10 mM DMPO in 0.10 M $HClO_4$ recorded on a 7 by 1 cm BDD electrode at 0.1 V s^{-1} before electrolysis (black), after a 5 min electrolysis +1.90 V vs SCE (red) and after a cleaning step (blue). Four cleaning methods were investigated: (a) rinsing the electrode with deionised water (b) polishing with alumina slurry (c) a cathodic treatment at -2.00 V vs SCE for 60 sec in 0.10 M $HClO_4$ and (d) an anodic treatment at +2.00 V vs SCE for 60 sec in 0.10 M $HClO_4$.

SI.4 Radical Concentration Calibration using 4-hydroxy TEMPO

A stable radical species in solution, 4-hydroxy-2,2,6,6-tetramethyl-1-piperidine 1-oxyl (4-hydroxy TEMPO) was prepared at 500 nM, 1 μM, 10 μM and 100 μM concentrations in order to produce a radical concentration calibration plot. Exemplar EPR spectra at 500 nM, 1 μM, 10 μM and 100 μM 4-hydroxy TEMPO concentrations are presented in **Figure S5.** Each spectrum is fitted with a simulated spectrum using EasySpin to extract the double-integrated for the known concentration of radical, which is proportional to the number of spins. These values are used to produce a calibration plot against which the DMPO spin adduct double integrated intensities can be compared to determine the concentration of DMPO spin adducts in solution. This calibration was performed everytime EPR data were collected in order to account for fluctations in the instrument.

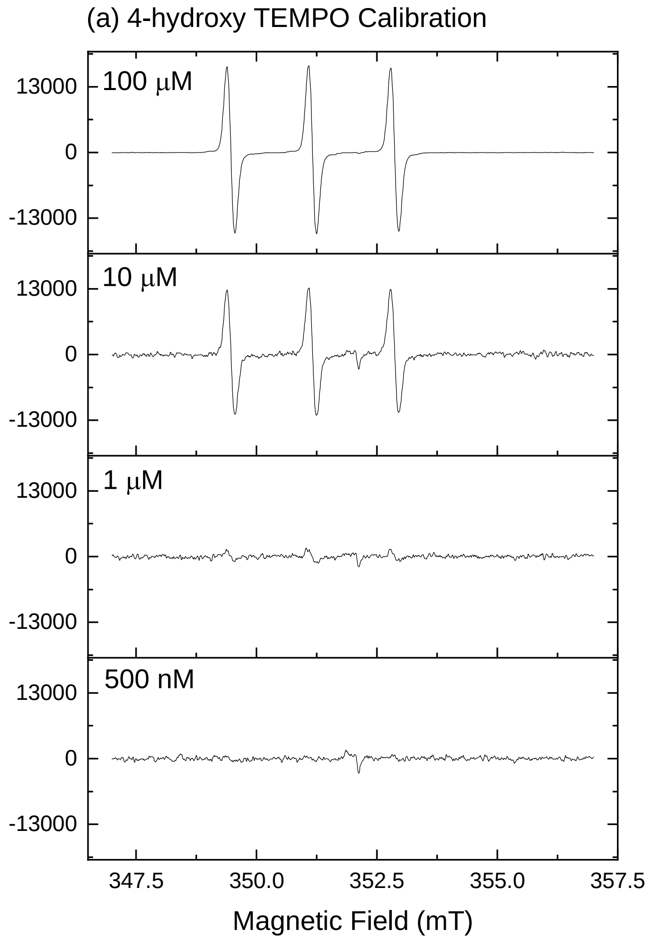

Figure S5: EPR spectra of 500 nM, 1 μM, 10 μM and 100 μM 4-hydroxy TEMPO.

SI.5 Estimation of Diffusion Coefficient for DMPO

The diffusion coefficient (*D*) for DMPO in 0.10 M $HClO_4$ has been estimated using the Wilke-Chang equation,[3] eq. S1.

$$D = 7.4 \times 10^{-8} \frac{((\varphi MW_s)^{\frac{1}{2}}T)}{\eta (V_a)^{0.6}} \qquad \text{(S1)}$$

φ is the factor for solute-solvent interaction (2.6 for water)[4], MW_s is the molecular weight of the solvent (for water, 18 g mol^{-1}), T is the absolute temperature (298 K), η is the dynamic viscosity of the solvent (0.89 mPa.s at 25 °C of water)[5] and V_a is the molar volume of the analyte. The molar volume of the analyte (cm^3 mol^{-1}) can be determined using eq. S2.

$$V_a = \frac{MW_a}{\rho_a} \qquad \text{(S2)}$$

where MW_a is the molecular weight of the analyte (for DMPO, 113.16 g mol^{-1}) and ρ_a is the density of the analyte (1.015 g cm^{-3}).

The molar volume of DMPO was calculated to be 111.49 cm^3 mol^{-1} and therefore, *D* for DMPO was estimated as 9.9×10^{-6} cm^2 s^{-1}.

SI.6 Digital Simulations

Digital simulations were performed using DigiElch (Version 8.0) to model the electron transfer (E) mechanism, eq. S3, followed by the chemical step (C), eq. S4. It is assumed that the electron transfer step is reversible i.e. sufficiently fast electron transfer rate constant (= 1×10^5 cm s^{-1}). Electron transfer only data are also shown. An effective rate constant describing the chemical steps (k_{sol}) is simulated for values from 0.1 to 1000 s^{-1}, **Figure S6**. Mass transport is assumed to be planar, semi-infinite 1D diffusion. The simulation used 6 Gauss-Newton Iterations and a truncation error of 1E-5% taking < 1 s to solve one voltammogram when the applied electrode potential was stepped in 1 mV increments. The diffusion coefficient of $DMPO^{+\cdot}$ is assumed to be equal to that of DMPO = 9.9×10^{-6} cm^2 s^{-1}.

$$DMPO \rightleftharpoons DMPO^{+\cdot} + e^- \qquad (E) \qquad (S3)$$

$$DMPO^{+\cdot} \rightarrow DMPO\text{-}OH^{\cdot} \qquad (C_{sol}) \qquad (S4)$$

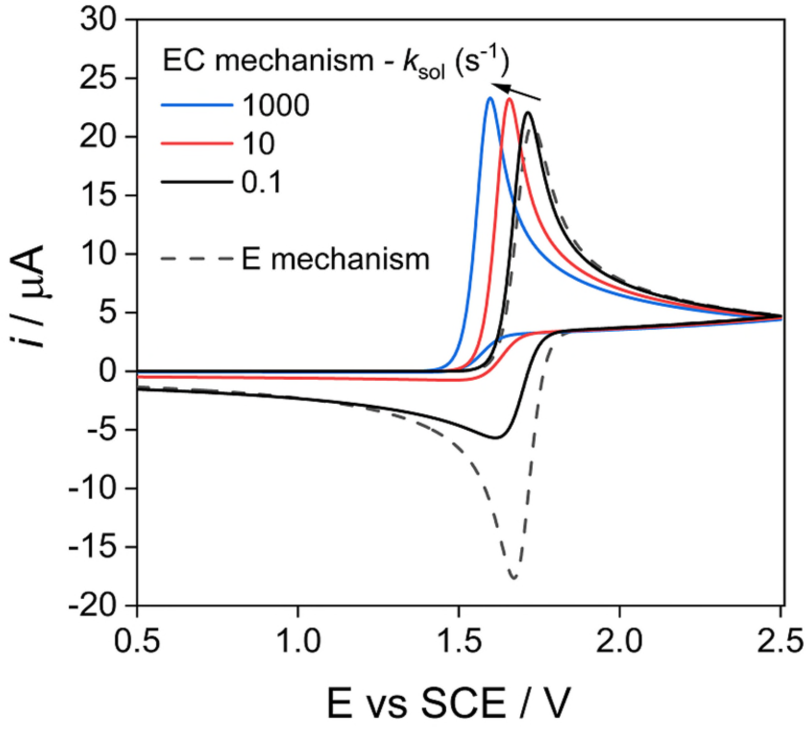

Figure S6: DigiElch simulations of the oxidation of DMPO, assuming an EC_{sol} process for solution rate constants of 0.1, 10 and 1000 s^{-1}. The complete set of experimental parameters are given in the main text of the paper. Also shown is the case for reversible E only.

SI.7 Electrochemical Oxidation Pathways of DMPO

Possible electron loss pathways of DMPO and its oxidation products are shown in **Figure S7**. Electrochemical oxidation of DMPO produces $DMPO^{+\cdot}$, which rapidly reacts with water to form the DMPO-OH$^{\cdot}$ spin adduct. DMPO-OH$^{\cdot}$ can then undergo a further one electron oxidation to form the tautomers: 1-hydroxy-5,5-dimethyl-1-pyrrolid-2-one (HDMPN) and 2-hydroxy-5,5-dimethyl-1-pyrroline-N-oxide (HDMPO) which are diamagnetic species and not observed in EPR. Each tautomer can undergo a further one electron oxidation to form either the stable paramagnetic species: 5,5-dimethyl-2-oxopyrroline-N-oxyl (DMPO-X$^{\cdot}$) or 2-dihydroxy-5,5-dimethyl-1-pyrrolidinyloxy (HDMPO-OH$^{\cdot}$).

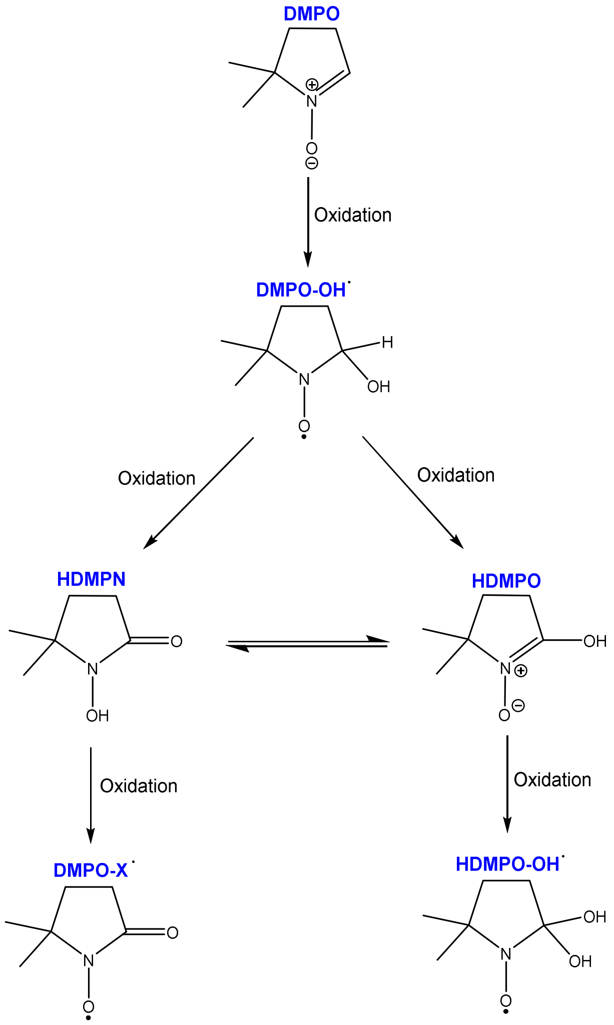

Figure S7: Reaction pathways for the electrochemical oxidation of DMPO and its oxidative products.

SI.8 Electrochemical-EPR Experiments at Higher Potentials

Figure S8 shows the EPR detection of DMPO-OH˙ as a function of applied electrode potential (vs SCE). From +0.90 V up to +2.90 V the potential is increased in steps of 0.20 V and from +3.00 V up to +5.00 V vs SCE, the step potential is changed to 0.5 V. The solution contained 10 mM DMPO in 0.10 M $HClO_4$. A cleaning pre-treatment of applying a cathodic potential (-2.00 V vs SCE for 60 s in 0.10 M $HClO_4$) was made before each new electrode measurement. Beyond +2.90 V vs SCE and up to a maximum of +5.00 V vs SCE, the signal for DMPO-OH˙ continues to drop off overall. Interestingly, no other paramagnetic products (DMPO-X˙ and HDMPO-OH˙ in **Figure S8a**) were detected even at these higher potentials.

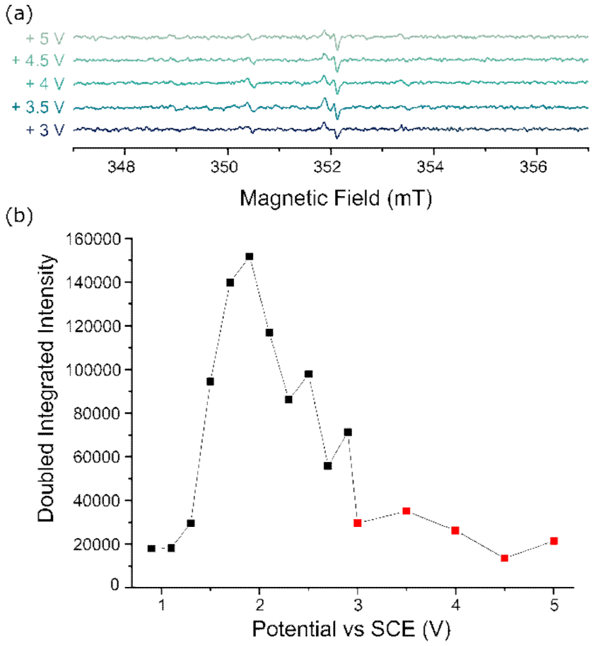

Figure S8: (a) EPR spectra for 5 min electrolysis of 10 mM DMPO in 0.10 M $HClO_4$ at potentials of +3.00 V, +3.50 V, +4.00 V, +4.50 V and +5.00 V vs SCE, using a 7 by 1 cm BDD electrode immersed to a depth of ca. 5 cm. (b) Double integrated intensity of DMPO-OH˙ as a function of potential. Values at the potentials of +0.90 V to +2.90 V vs SCE are taken from **Figure 3** (main text). New data points at +3.00, +3.50, +4.00, +4.50 and +5.00 V vs SCE are highlighted in red.

SI.9 Solvent Window of 0.1 M TBAB in Ethanol

To determine at which potential ethanol is oxidized, a solvent window for 0.10 M tetrabutylammonium tetrafluoroborate (TBAB) in ethanol was recorded on a 1 mm diameter disk BDD electrode, at 0.1 V s^{-1}. Note, the scan commences at +0.00 V vs SCE then first proceeds in an anodic direction.

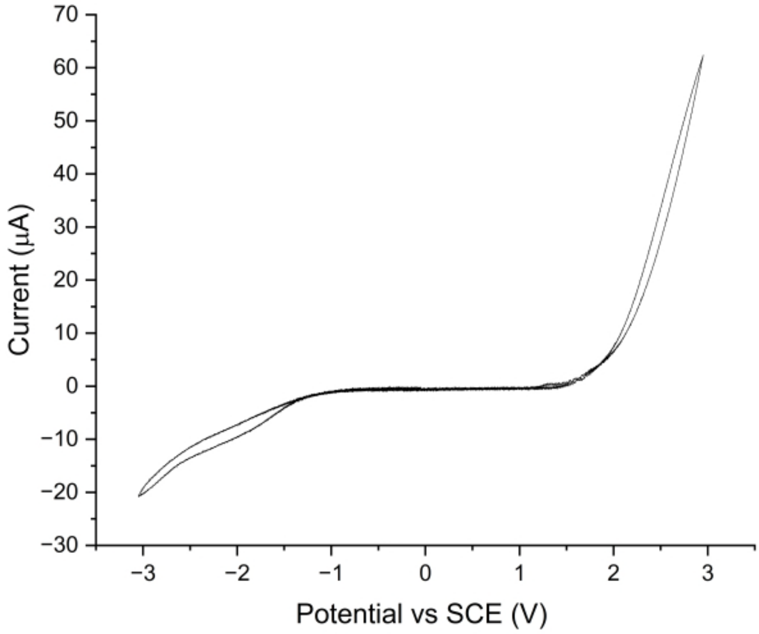

Figure S9: First scan CV of 0.10 M TBAB in ethanol on 1 mm diameter disk BDD macroelectrode at 0.1 V s^{-1} vs SCE.

SI.10 Mechanism for Electrochemical Oxidation of MNP, PBN and POBN

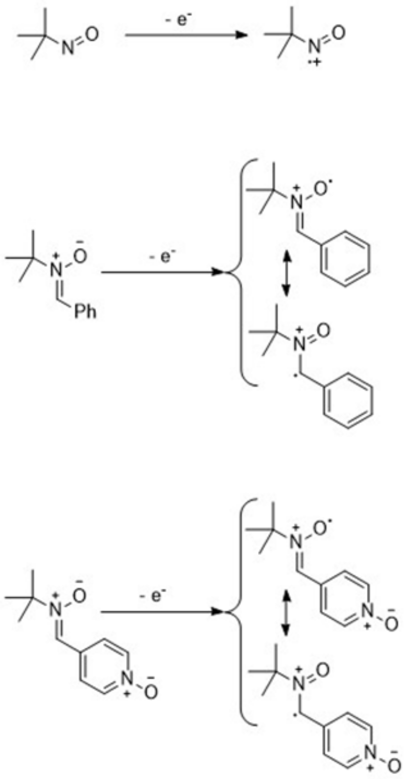

Figure S10: Mechanism for the electrochemical oxidation of MNP, PBN and POBN (top to bottom respectively).

References

(1) Macpherson, J. V. A Practical Guide to Using Boron Doped Diamond in Electrochemical Research. *Phys. Chem. Chem. Phys.* **2015**, *17*, 2935–2949. https://doi.org/10.1039/c4cp04022h.

(2) Hutton, L. A.; Iacobini, J. G.; Bitziou, E.; Channon, R. B.; Newton, M. E.; Macpherson, J. V. Examination of the Factors Affecting the Electrochemical Performance of Oxygen-Terminated Polycrystalline Boron-Doped Diamond Electrodes. *Anal. Chem.* **2013**, *85* (15), 7230–7240. https://doi.org/10.1021/ac401042t.

(3) Wilke, C. R.; Chang, P. Correlation of Diffusiong Coefficients in Dilute Solutions. *A.I.Ch.E. J.* **1955**, *1* (2), 264–270.

(4) Miyabe, K.; Isogai, R. Estimation of Molecular Diffusivity in Liquid Phase Systems by the Wilke – Chang Equation. **2011**, *1218*, 6639–6645. https://doi.org/10.1016/j.chroma.2011.07.018.

(5) Calculator, O. Water Viscosity Calculator https://www.omnicalculator.com/physics/water-viscosity.
